# Supplementary material for: Differential Effect of Free-Air CO2 Enrichment (FACE) in Different Organs and Growth Stages of Two Cultivars of Durum Wheat
Source: Plants (Basel). 2023 Feb 3;12(3):686. doi: 10.3390/plants12030686 (PMC9920850; doi:10.3390/plants12030686)
Supplement: Supplementary file 1 [file plants-12-00686-s001.zip › plants-2167417-supplementary.pdf]

plants

Supplementary:

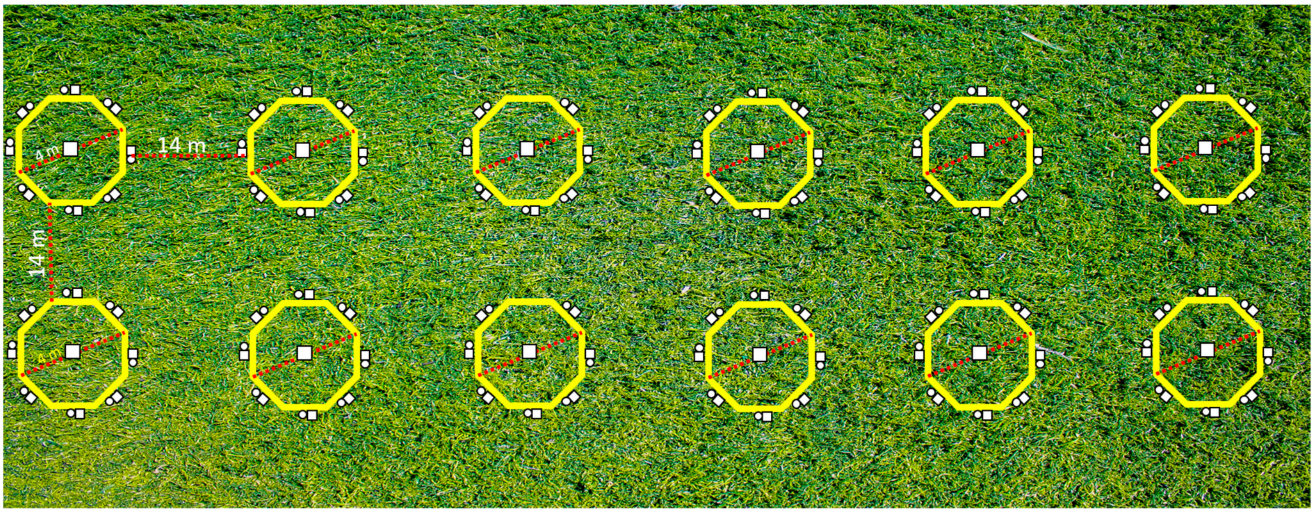

Figure S1. Design of mini-FACE experimental showing distribution of rings into field. Source: Own elaboration.
